# Open exchange of scientific knowledge and European copyright: The case of biodiversity information

**DOI:** 10.3897/zookeys.414.7717

**Published:** 2014-06-06

**Authors:** Willi Egloff, David J. Patterson, Donat Agosti, Gregor Hagedorn

**Affiliations:** 1 Plazi, Zinggstrasse 16, 3007 Berne, Switzerland; 2 Museum für Naturkunde, Invalidenstrasse 43, 10115 Berlin, Germany

**Keywords:** Biodiversity knowledge, taxonomy, intellectual property rights, European copyright, European database protection right, data use agreement, Open Access to data and information

## Abstract

**Background.** The 7^th^ Framework Programme for Research and Technological Development is helping the European Union to prepare for an integrative system for intelligent management of biodiversity knowledge. The infrastructure that is envisaged and that will be further developed within the Programme “Horizon 2020” aims to provide open and free access to taxonomic information to anyone with a requirement for biodiversity data, without the need for individual consent of other persons or institutions. Open and free access to information will foster the re-use and improve the quality of data, will accelerate research, and will promote new types of research. Progress towards the goal of free and open access to content is hampered by numerous technical, economic, sociological, legal, and other factors. The present article addresses barriers to the open exchange of biodiversity knowledge that arise from European laws, in particular European legislation on copyright and database protection rights.

We present a legal point of view as to what will be needed to bring distributed information together and facilitate its re-use by data mining, integration into semantic knowledge systems, and similar techniques. We address exceptions and limitations of copyright or database protection within Europe, and we point to the importance of data use agreements. We illustrate how exceptions and limitations have been transformed into national legislations within some European states to create inconsistencies that impede access to biodiversity information.

**Conclusions.** The legal situation within the EU is unsatisfactory because there are inconsistencies among states that hamper the deployment of an open biodiversity knowledge management system. Scientists within the EU who work with copyright protected works or with protected databases have to be aware of regulations that vary from country to country. This is a major stumbling block to international collaboration and is an impediment to the open exchange of biodiversity knowledge. Such differences should be removed by unifying exceptions and limitations for research purposes in a binding, Europe-wide regulation.

## Introduction

Biodiversity data and information are important sources of knowledge for many biological, geological, and environmental research disciplines as well as for the development of policies relating to the natural environment and the management of natural resources (National Research Council 2009). The core knowledge about biodiversity has accumulated over hundreds of years in taxonomic literature, in natural history institutions, herbaria, botanic gardens; through ongoing biodiversity monitoring programs; and increasingly through digital initiatives.

Scientific names of organisms are an important component of biodiversity information. They act as a tally of how much biodiversity has been described. They are universally used to index biodiversity information (Patterson et al. 2010). Other sources of biodiversity information include hierarchical classifications of taxa; nomenclators and registries compiling information about names in the context of codes of nomenclature; taxonomic treatments being those parts of publications that include nomenclatural or taxonomic acts such as descriptions of species; checklists listing names in a particular context; and monographs or other taxonomic revisions that re-evaluate our understanding of biodiversity within a particular domain. In addition to scientific names, their origins, etymologies, histories, synonymies, and authorships, such sources contain descriptions of taxa, hierarchical classifications, information about specimens, citations of literature, reference to images and other media with information about species, information on distribution, ecology, uses, common names, conservation status, evolutionary history, and other aspects of the biology of species (Patterson et al. 2014).

The infrastructure for a system that will intelligently manage and integrate digital biodiversity information - an Open Biodiversity Knowledge Management System (OBKMS) - will need access to all names, taxonomic information, and other biodiversity data in formats that can be understood by both humans and computers (Dulong De Rosnay and Guadamuz 2013). If we are to realise the vision of Open Biodiversity Knowledge Management (OBKM), access to and re-use of data and information will need to be open, free, and independent of any individual authorisation by other persons or institutions. The system must allow intended and expected re-uses as well as unforeseen, innovative re-uses of the information.

The project “pro-iBiosphere”, financed through the European 7^th^ Framework Programme for Research and Technological Development, and in the perspective of the EU Framework Programme for Research and Innovation “Horizon 2020”, has evaluated the requirements for an OBKMS that will: (i) offer a robust service-oriented architecture suited to working with taxon-level information distributed at multiple sources; (ii) have a central registry of sources and services to help users find available data and services and to understand how to use them; (iii) provide open and free access to all names and taxonomic information for anyone with a requirement for biodiversity data, without depending on consent of other individuals or institutions; and (iv) facilitate the re-use of biodiversity data and information (pro-iBiosphere 2012).

At present, the access to and re-use of biodiversity data is hampered by an array of technical, economic, sociological, legal and other factors (Thessen and Patterson 2011). Many compilers of taxonomic content act as if or claim that they hold intellectual property rights over their data and information (Patterson et al. 2014), erecting legal barriers for access and re-use. Open and free access to biodiversity data and information will require a different attitude: there should be no legal provisions related to copyright that may prevent anyone whose activity requires biodiversity data from freely accessing and re-using such data from the place and at the time of their choice as well as in the technical form they prefer. This article addresses legal factors in Europe that will have to be overcome if we are to build an OBKMS.

## The request for open access

The political agenda for open access started with the Ministerial declaration of the high-level segment of the United Nations Economic and Social Council of 2000 “Development and international cooperation in the twenty-first century: the role of information technology in the context of a knowledge-based global economy” (United Nations 2000). In section 15, the declaration outlines “the importance of universal access to knowledge and information for promoting development”. This rather general assertion is re-expressed by the Budapest Open Access Initiative, which defines open access as the “free availability of scientific literature on the public internet, permitting any users to read, download, copy, distribute, print, search, or link to the full texts of these articles, crawl them for indexing, pass them as data to software, or use them for any lawful purpose, without financial, legal, or technical barriers other than those inseparable from gaining access to the internet itself” (Budapest Open Access Initiative 2001). The concept has been further developed in the 2003 Berlin Declaration (Berlin Declaration on Open Access to Knowledge in the Sciences and Humanities 2003). Open Access stresses the desirability of equitable access to knowledge across an economically and socially uneven world. The principle of open access to knowledge and information has been reaffirmed in numerous discipline-based, national, or international statements and declarations (Open Access Directory 2013). It is particularly important in sciences because of the importance of sharing data and information from which scientific insights are developed.

Open access should apply to the results of scientific research - such as raw data and metadata, source materials, processed data, results of analyses, and pictures, graphs and other diagrams derived from and representing the data. Open access should apply to every form of scholarly publication and other contributions to scientific knowledge. In terms of the Berlin Declaration, open access contributions need to satisfy at least the following two conditions:

1. “The author(s) and right holder(s) of such contributions grant(s) to all users a free, irrevocable, worldwide, right of access to, and a license to copy, use, distribute, transmit and display the work publicly and to make and distribute derivative works, in any digital medium for any responsible purpose, subject to proper attribution of authorship (…), as well as the right to make small numbers of printed copies for their personal use.

2. A complete version of the work and all supplemental materials, including a copy of the permission as stated above, in an appropriate standard electronic format is deposited (and thus published) in at least one online repository using suitable technical standards (…) that is supported and maintained by an academic institution, scholarly society, government agency, or other well-established organisation that seeks to enable open access, unrestricted distribution, interoperability, and long-term archiving” (Berlin Declaration on open access to knowledge in the sciences and humanitites 2003).

In the “Joint Declaration on Open Science for the 21st Century”, presented by the European Federation of Academies of Sciences and Humanities and the European Commission on 11th April, 2012, the vision of Open Science is summarised as follows:

“Open Science envisages optimal sharing of research results and tools: publications, data, software, and educational resources. It will rely on advanced e-infrastructures that enable online research collaboration. The potential to link cognate, and to re-use initially unrelated datasets will reveal unexpected relationships and will trigger new dynamics of scientific discovery. The collective intelligence of scientific communities will be unleashed through new collaborations across institutional, disciplinary, sectoral and national boundaries. The open science environment will help restore transparency and integrity to the scientific enterprise, for all to see. New points of exchange with non-academic end-users of scientific knowledge will be created, and progress will be made towards the vision of scientifically literate societies: this may require releasing scientific data in forms that are accessible to citizens” (ALLEA 2012).

In order to reach that goal, scientific publications must be made openly available, as soon after publication and as freely as possible. Restrictions should be based on specific justifications, such as to protect security, endangered species, or to protect the privacy of individuals. As a first step, the principles of open science should be applied to all content arising from – fully or partially – publicly funded research. Research proposals requesting public funds should include measures aimed at advancing open science and apply open access principles. Further steps will be necessary in order to extend these principles to all scientific research of general societal interest. A discussion, however, of how to balance commercial interests with societal interests is beyond the topic of the present contribution.

## Why copyright can hamper the exchange of biodiversity knowledge

Copyright protects works that are original, individual, new creations with respect of their form of presentation. It gives the owner the exclusive right to reproduce the work, to distribute it, to communicate it to the public, to make derivative versions, to transfer rights to others as well as several other rights (WIPO 1979). Copyright does not protect content in itself; the protection refers to the form of expression. Copyright protection applies only if the content is expressed in an original way. If content is presented in a pre-existing, familiar or standardised form, it does not qualify as work in the sense of copyright.

Scientific names of species follow a standardised format that was developed more than 250 years ago. This includes the latinisation of words and the use of a binomial name for species. Even when a name is new, the form of expression follows the well-established pattern. The same can be said about taxonomic treatments and much other taxonomic information which is expressed in standardised forms and language. The familiar nature of the information is valuable as it helps other scientists to understand the treatments, to compare them, and to draw their content together in their own scientific work. The familiar nature means that the material cannot be subject to copyright. Elements of taxonomic information that are ‘familiar’ to taxonomists were summarised as a ‘blue list’ that included: scientific names; etymology of names, authorities for names, nomenclatural information and opinions, rank and/or hierarchical organisation or classification; alphabetical, chronological, phylogenetic, palaeontological, geographical, ecological, host-based, or feature-based ordering of taxa; synonyms and taxonomic opinions; references to relevant literature, type material, and images; data about materials examined; information on overall distribution, ecology, conservation and uses and descriptions of the taxon (Patterson et al. 2014).

None-the-less, copyright statements are often associated with taxonomic information - especially on web sites. These refer to ownership and address the re-use of content. Most of these statements lack legal foundation because the data and information to which they refer do not qualify as works in the sense of copyright legislation. There are no legitimate copyright restrictions on the re-use of such information. A non-copyrightable publication remains non-copyrightable even if the author or others choose to mark it with a copyright statement, a copyright mark (such as ©), or with a Creative Commons license.

Biodiversity data and information are rarely presented as unembellished flat lists. They are parts of websites, articles, monographs, and other forms of publications. Even if the data or information are not copyrightable, the website or monograph as a whole may qualify as work and therefore be copyright protected. If the process of re-use requires the reproduction of copyright protected parts of the source, then authorisation of the copyright holder is required (Agosti and Egloff 2009).

European copyright legislation is well aware of the fact that copyright may present a barrier to scientific work. The EU-Directive 2001/29/EC on the harmonisation of certain aspects of copyright and related rights in the information society (European Parliament and the Council of the European Union 2001) addresses the challenge and puts considerable weight on the importance of science by providing for exceptions and limitations to copyright. It grants to the author the rights to decide who shall be allowed to reproduce his work (“reproduction right”) or who shall be allowed to communicate it to the public (“communication right”), but it provides for restrictions (“exceptions and limitations”) to these rights in the general interest. Recital 34 to the Directive provides for exceptions and limitations for “educational and scientific purposes”. Recital 40 refers to exceptions and limitations “for the benefit of certain non-profit making establishments such as publicly accessible libraries and equivalent institutions, as well as archives”. Directive 2001/29/EC therefore gives member states the option of establishing their own laws for the following exceptions and limitations:

Article 5.2.

Member States may provide for exceptions and limitations to the reproduction right provided for in Article 2 in the following cases:

(…)

c) in respect of specific acts of reproduction made by publicly accessible libraries, educational establishments or museums, or by archives, which are not for direct or indirect economic or commercial advantage.

(…)”

Article 5.3

Member States may provide for exceptions and limitations to the rights provided for in Article 2 and 3 in the following cases:

a) use for the sole purpose of illustration for teaching or scientific research, as long as the source, including the author’s name, is indicated, unless this turns out to be impossible and to the extent justified by the non-commercial purposes to be achieved;

(…)

n) use by communication or making available, for the purpose of research or private study, to individual members of the public by dedicated terminals on the premises of establishments referred to in paragraph 2(c) of works and other subject-matter no subject to purchase or licensing terms which are contained in their collections;

(…)”

These exceptions and limitations are only applicable when they are transformed into national law by individual member states of the EU. Then, they apply only to that member state. Such transformations result in copyright provisions that differ from country to country. Therefore, scientists who rely on data from different EU member states or who collaborate internationally need to be aware that different legal frameworks may apply to the data they use. In the Communication on “Copyright in the Knowledge Economy”, the EU Commission makes it clear that this situation is a major stumbling block to international scientific cooperation within the EU and declares that “limiting teaching and research to a specific location is considered to be contrary to the realities of modern life” (European Commission 2009).

In the context of the “Licenses for Europe – structured stakeholder dialogue 2013” (European Commission 2013), the European Commission established a special working group to discuss adaptations of the copyright framework regarding text and data mining (TDM). The Commission’s objective was to promote the efficient use of TDM for scientific research purposes. As TDM currently requires contractual agreements between users (e.g. research institutions) and rights holders (e.g. publishers of scientific journals) to establish the terms and conditions for technical access to the relevant data sets, the working group explored solutions such as standard licensing models as well as the design of technology platforms to facilitate TDM. The working group was unable to complete its mandate. Ten organisations representing interests within the research community concluded that progress was made impossible by the attitude of publishers who insisted on licensing models as the only acceptable solution and left the group.

## The European database protection

In 1996, the European Union introduced a special legal protection for databases (European Parliament and the Council of the European Union 1996). It applies to databases which show “that there has been qualitatively and/or quantitatively a substantial investment in either the obtaining, verification or presentation of the contents” (art. 7 of the Directive 96/9/EC). It allows owners to prevent extraction and/or re-use of the whole or of a substantial part, evaluated qualitatively and/or quantitatively, of the content of a database. The concept is unique to the EU. No similar rights exist in the U.S. or in European non-member states of the EU or the EEA.

As the European Court of Justice pointed out in several judgments, database protection concerns the creation of databases out of material that already exists, but does not deal with the creation of data as such (European court of Justice 2004a). The expression “investment in the obtaining of the contents” refers therefore to the resources used to find existing materials and collect them in the database, and not to the resources used to create materials. The expression “investment in the verification of the contents” refers to the resources used to monitor the accuracy of the materials collected when the database was created and during its operation, but does not refer to the resources used for verification during the creation of materials. The expression “investment in the presentation of the contents” concerns the resources used to process and deliver information (European Court of Justice 2004b).

According to the 48^th^ recital of the preamble to Directive 96/9/EC, database protection has an economic justification, being to afford protection to the maker of a database and guarantee a return on the investment in the creation and maintenance of the database. “Investment” refers only to the investment of private or commercial resources, but not to the use of public funding. In consequence, the protection applies exclusively to private databases. As most databases with relevant taxonomic information are mainly publicly funded, database protection for them is quite limited. However, there are databases containing biodiversity data which are protected by this so called “sui-generis-right”.

Database protection does not deal with individual data elements, but with databases as a whole. Database protection concerns the creation of databases but does not deal with the protection of data as such. It only prevents the extraction and re-utilisation of substantial parts of a database. Therefore, it cannot impede the access to a database, but only, in very particular circumstances, restrict the re-use of the whole or a substantial part of a database.

According to Art. 9 (b) of the Directive 96/9/EC, member states may stipulate that lawful users of a database may extract or re-utilise a substantial part of its contents for the purposes of scientific research. This exception is only applicable if transformed into national law by individual member states of the EU. Therefore, scientists in different EU member states have to be aware of different legal frameworks as far as database legislation is concerned.

## The importance of data use agreements

Access to and re-use of biodiversity data and information is not only regulated by copyright and database protection, but also through individual data use agreements (DUA). Such agreements can relate to data and information irrespective of whether copyright or databases rights apply. They can have the form of a written contact, or be a general “terms of use”-statement which the user has to accept before accessing protected data and information. Indeed, a DUA may simply be said to have been agreed as a result of accessing content. Such agreements are frequently called “licenses” although the term is, in many cases, inappropriate from a legal point of view.

A DUA is only valid if both parties have accepted the conditions of access or terms of use, and applies only to those parties. In many cases, it may be difficult to decide if there is an agreement or not and if a potential licensee has accepted it. An agreement requires an active expression of intent, whether spoken, written, or in fact. The mere action of accessing data and information on a website that is accompanied by a “terms of use”-statement or any similar declaration, will rarely be considered as consent.

Creative Commons licenses or similar are a form of data use agreement. They are declared unilaterally by a “licensor”, and they are valid under the condition that the licensee has accepted them. Their importance lies in the fact that they often declare a restriction to copyright and, with the new version 4, to database protection: The licensor declares to any interested user that he or she will not claim copyright or database rights as long as the material is re-used in a way that is explicitly authorised by the CC-license. Such terms of use appeal to licensors and licensees as it increases access to and use of works and databases.

In Europe, data use agreements can regulate the access to and the re-use of copyrighted works or protected databases only when there is no binding legal regulation in form of exceptions and limitations to copyright or database protection. The European Court of Justice ruled in a judgment of June 23, 2013, as follows: “Where a Member State has decided, pursuant to a provision in Article 5(2) and (3) of Directive 2001/29, to exclude, from the material scope of that provision, any right for the rightholders to authorise reproduction of their protected works or other subject-matter, any authorising act the rightholders may adopt is devoid of legal effects under the law of that State” (European Court of Justice 2013). Binding legal exceptions and limitations that allow certain uses of copyright protected works or of databases supersede any conflicting data use agreement, including Creative-Common-licenses. Terms and conditions that contradict such legal regulations are void, as is clearly expressed for example in section 29A, par. 5, and section 50 D, par. 2, of the British CPDA, cited in the next section below. However, data use agreements can supersede legal regulations where this possibility is provided for in the same legal text, as in the cases, for example, of § 52b and 53a of the German copyright law or in Art. 71-ter of the Italian Copyright law, cited in the next section.

## Examples of national regulations

Exceptions and limitations to copyright and database protection apply if transformed into national law of member states of the European Union or the European Economic Area. The resulting national legislation differs from country to country (Dietrich et al. 2013). The following list illustrates the resulting diversity. The examples have been chosen with respect to: a) the special legal situations that are found in some countries (case law regulation in the United Kingdom, extended collective licenses in the Scandinavian countries, specific database regulations in Germany and Italy); b) the economic weight in the sector of scholarly publishing (United Kingdom, Germany, France); or c) the political status of the country (by including non-EU-members such as Norway and Switzerland).

### Denmark

Copyright and database protection in Denmark is provided for by the “LBK nr 202 af 27/02/2010 Gældende (Ophavsretsloven)” (Danske retsinformation 2010). The exceptions and limitations provided for in Directive 2001/29/EC und Directive 96/9/EC have in part been transformed into national law in the form of extended collective licenses (“aftalelicenser”). An extended collective license is an agreement between a qualified user (archive, libraries, and others) and an officially recognised collecting agency which represents “an essential part of authors whose works are used in Denmark” (§ 50). The following articles are particularly relevant:

§ 13 (referring to article 5.3.a of Directive 2001/29/EC):

Til brug i undervisningsvirksomhed kan der fremstilles eksemplarer af udgivne værker samt ved optagelse foretages eksemplarfremstilling af værker, som udsendes i radio eller fjernsyn, såfremt betingelserne for aftalelicens efter § 50 er opfyldt. (…)

§ 14 (referrring to internal use in institutions and enterprises):

Offentlige eller private institutioner, organisationer og erhvervsvirksomheder kan til intern brug i deres virksomhed ved fotokopierung eller lignende fremstille eller lade fremstille eksemplarer af fagmæssige artikler i aviser, tidsskrifter og samleværker, af korte afsnit af andre udgivne værker af fagmæssig art, af musikværker samt af illustrationer, som er gengivet i tilslutning til teksten, såfremt betingelserne for aftalelicens efter § 50 er opfyldt. Sådanne eksemplarer må kun udnyttes inden for virksomhed, som omfattes af den i § 50 forudsatte aftale. (…)

§ 16 (referring to article 5.2.c of Directive 2001/29/EC):

^1^Offentlige arkiver, offentlige biblioteker og andre biblioteker, der helt eller delvis finanseres af det offentlige, samt statslige museer og museer, der er godkendt efter museumsloven, må gengive og sprede eksemplarer af værker til brug i deres virksomhed i overensstemmelse med bestemmelserne i stk. 2-6, såfremt det ikke sker i erhvervsøjemed. Dette gælder dog ikke for edb-programmer i digital form bortset fra computerspil.

^2^Institutionerne må fremstille eksemplarer i sikkerheds- og beskyttelsesøjemed.

^3^Såfremt et eksemplar i en institutions samling er ufuldstændigt, må institutionen fremstille eksemplarer af de manglende dele, medmindre værket kan erhverves i almindelig handel eller hos udgiveren.

^4^Biblioteker kan fremstille eksemplarer af udgivne værker, der bør være tilgængelige i bibliotekets samlinger, men som ikke kan erhverves i almindelig handel eller hos udgiveren. (…)

§ 16a (referring to article 5.3.n of Directive 2001/29/EC):

Offentliggjorte værker kan gøres tilgængelige for enkeltpersoner på de i § 16, stk. 1, nævnte institutioner til personligt gennemsyn eller studium på stedet ved hjælp af teknisk udstyr. (…)

§ 16b (referring to article 5.2.c and 5.3.n of Directive 2001/29/EC):

Offentlige biblioteker og andre biblioteker, der helt eller delvis finansieres af det offentlige, kan på bestilling i digital form gengive artikler fra aviser, tidskrifter og samleværker, kortere afsnit af bøger og andre udgivne litterære værker samt illustrationer og noder, som er gengivet i tilslutning til teksten, såfremt betingelserne for aftalelicens efter § 50 er opfyldt. Bestemmelsen i 1. pkt. omfatter ikke udsendelse i radio eller fjernsyn eller tilrådighedsstillelse af værker på en sådan måde, at almenheden får adgang til dem på et individuelt valgt sted og tidspunkt (…).

§ 50 (referring to extended collective licenses):

^1^Aftalelicens efter §§ 13, 14 og § 16 b, § 17, stk. 4, og §§ 24, 30, 30 a og 35 kan påberåbes af brugere, der har indgået en aftale om den pågældende værksudnyttelse med en organisation, som omfatter en væsentlig del of ophavsmændene til en bestemt art af værker, der anvendes i Danmark.

^2^Aftalelicens kan desuden påberåbes af brugere, der inden for et nærmere defineret område har indgået aftale om værksudnyttelse med en organisation, der omfatter en væsentlig del of ophavsmændene til en bestemt art of værker, der anvendes i Danmark på det pågældende område. Dette gælder dog ikke, hvis ophavsmanden over for nogen af de aftalesluttende partner har nedlagt forbud mod værkets udnyttelse.

^3^Aftalelicensen giver brugeren ret til at udnytte andre værker af samme art, selv om ophavsmændene til disse værker ikke repræsenteres af organisationen. Aftalelicensen giver brugeren ret til at benytte de ikke-repræsenterede ophavsmænds værker på den måde og på de vilkår, som følger af den aftale, der er indgået med organisationen.

### France

Copyright and database protection in France is provided for by the 1^st^ part of the “Code de la propriété intellectuelle (L. n° 92-597 du 1er juillet 1992)” (République Française 1992). The exceptions and limitations provided for in Directive 2001/29/EC and Directive 96/9/EC have been transformed into the following articles:

Art. L 122-5-8° (referring to article 5.2.c of Directive 2001/29/EC):

Lorsque l’oeuvre a été divulguée, l’auteur ne peut interdire:

(…)

8°La reproduction d’une œuvre et sa représentation effectuées à des fins de conservation ou destinées à préserver les conditions de sa consultation à des fins de recherche ou d’études privées par des particuliers, dans les locaux de l’établissement et sur des terminaux dédiés par des bibliothèques accessibles au public, par des musées ou par des services d’archives, sous réserve que ceux-ci ne recherchent aucun avantage économique ou commercial;

(…)

Art. L 122-5-3° (referring to paragraph 5.3.a of Directive 2001/29/EC):

Lorsque l’oeuvre a été divulguée, l’auteur ne peut interdire:

(…)

3°Sous réserve que soient indiqués clairement le nom de l’auteur et la source:

(…)

e) La représentation ou la reproduction d’extraits d’œuvres, sous réserve des œuvres conçues à des fins pédagogiques, des partitions de musique et des œuvres réalisés pour une édition numérique de l’écrit, à des fins exclusives d’illustration dans le cadre de l’enseignement et de la recherche, à l’exclusion de toute activité ludique ou récréative, dès lors que le public auquel cette représentation ou cette reproduction est destinée est composé majoritairement d’élèves, d’étudiants, d’enseignants ou de chercheurs directement concernées, que l’utilisation de cette représentation ou cette reproduction ne donne lieu à aucune exploitation commerciale (…)

### Germany

Copyright and database protection in Germany is provided for by the “Gesetz über Urheberrecht und verwandte Schutzrechte (Urheberrechtsgesetz – UrhG) vom 9. September 1965“ (Bundesministerium der Justiz und für Verbraucherschutz 2013). The exceptions and limitations provided for in Directive 2001/29/EC and Directive 96/9/EC have been transformed into the following articles:

§ 52b UrhG (referring to paragraph 5.3.n of Directive 2001/29/EC):

Zulässig ist, veröffentlichte Werke aus dem Bestand öffentlich zugänglicher Bibliotheken, Museen oder Archive, die keinen unmittelbar oder mittelbar wirtschaftlichen oder Erwerbszweck verfolgen, ausschliesslich in den Räumen der jeweiligen Einrichtung an eigens dafür eingerichteten elektronischen Leseplätzen zur Forschung und für private Studien zugänglich zu machen, soweit dem keine vertraglichen Regeln entgegenstehen. Es dürfen grundsätzlich nicht mehr Exemplare eines Werkes an den eingerichteten elektronischen Leseplätzen gleichzeitig zugänglich gemacht werden, als der Bestand der Einrichtung umfasst. Für die Zugänglichmachung ist eine angemessene Vergütung zu zahlen. (…)

§ 52a UrhG (referring to paragraph 5.3.a of Directive 2001/29/EC):

(1) Zulässig ist,

1. veröffentlichte kleine Teile eines Werkes, Werke geringen Umfangs sowie einzelne Beiträge aus Zeitungen oder Zeitschriften zur Veranschaulichung im Unterricht an Schulen, Hochschulen, nichtgewerblichen Einrichtungen der Aus- und Weiterbildung sowie an Einrichtungen der Berufsbildung ausschliesslich für den bestimmt abgegrenzten Kreis von Unterrichtsteilnehmern oder

2. veröffentlichte Teile eines Werkes, Werke geringen Umfangs sowie einzelne Beiträge aus Zeitungen oder Zeitschriften ausschliesslich für einen bestimmt abgegrenzten Kreis von Personen für deren eigene wissenschaftliche Forschung

öffentlich zugänglich zu machen, soweit dies zu dem jeweiligen Zweck geboten und zur Verfolgung nicht kommerzieller Zwecke gerechtfertigt ist.

(2) Die öffentliche Zugänglichmachung eines für den Unterrichtsgebrauch an Schulen bestimmten Werkes ist stets nur mit Einwilligung des Berechtigten zulässig. Die öffentliche Zugänglichmachung eines Filmwerkes ist vor Ablauf von zwei Jahren nach Beginn der üblichen regulären Auswertung in Filmtheatern im Geltungsbereich dieses Gesetzes stets nur mit Einwilligung des Berechtigten zulässig.

(…)

§53 UrhG (referring to paragraph 5.3.a of Directive 2001/29/EC):

(1) Zulässig sind einzelne Vervielfältigungen eines Werkes durch eine natürliche Person zum privaten Gebrauch auf beliebigen Trägern, sofern sie weder unmittelbar noch mitttelbar Erwerbszwecken dienen, soweit nicht zur Vervielfältigung eine offensichtlich rechtswidrig hergestellte oder öffentlich zugänglich gemachte Vorlage verwendet wird. Der zur Vervielfältigung Befugte darf die Vervielfältigungsstücke auch durch einen anderen herstellen lassen, sofern dies unentgeltlich geschieht oder es sich um Vervielfältigungen auf Papier oder einem andern ähnlichen Träger mittels beliebiger photomechanischer Verfahren oder anderer Verfahren mit ähnlicher Wirkung handelt.

(2) Zulässig ist, einzelne Vervielfältigungsstücke eines Werkes herzustellen oder herstellen zu lassen

1. zum eigenen wissenschaftlichen Gebrauch, wenn und soweit die Vervielfältigung zu diesem Zweck geboten ist und sie keinen gewerblichen Zwecken dient,

2. (…)

(3) Zulässig ist, Vervielfältigungsstücke von kleinen Teilen eines Werkes, von Werken von geringem Umfang oder von einzelnen Beiträgen, die in Zeitungen oder Zeitschriften erschienen und öffentlich zugänglich gemacht worden sind, zum eigenen Gebrauch

1. zur Veranschaulichung des Unterrichts in Schulen, in nichtgewerblichen Einrichtungen der Aus- und Weiterbildung sowie in Einrichtungen der Berufsbildung in der für die Unterrichtsteilnehmer erforderlichen Anzahl oder

2. für staatliche Prüfungen und Prüfungen in Schulen, Hochschulen, in nichtgewerblichen Einrichtungen der Aus- und Weiterbildung sowie in der Berufsbildung in der erforderlichen Anzahl

herzustellen oder herstellen zu lassen, wenn und soweit die Vervielfältigung zu diesem Zweck geboten ist. Die Vervielfältigung eines Werkes, das für den Unterrichtsgebrauch bestimmt ist, ist stets nur mit Einwilligung des Berechtigten zulässig.

(…)

§53a (referring to paragraph 5.3.n of Directive 2001/29/EC):

(1) Zulässig ist auf Einzelbestellung die Vervielfältigung und Übermittlung einzelner in Zeitungen und Zeitschriften erschienener Beträge sowie kleiner Teile eines erschienenen Werkes im Wege des Post- oder Faxversands durch öffentliche Bibliotheken, sofern die Nutzung durch den Besteller nach § 53 zulässig ist. Die Vervielfältigung und Übermittlung in sonstiger elektronischer Form ist ausschliesslich als grafische Datei und zur Veranschaulichung des Unterrichts oder für Zwecke der wissenschaftlichen Forschung zulässig, soweit dies zur Verfolgung nicht gewerblicher Zwecke gerechtfertigt ist. Die Vervielfältigung und Übermittlung in sonstiger elektronischer Form ist ferner nur dann zulässig, wenn der Zugang zu den Beiträgen oder kleinen Teilen eines Werkes den Mitgliedern der Öffentlichkeit nicht offensichtlich von Orten und Zeiten ihrer Wahl mittels einer vertraglichen Vereinbarung zu angemessenen Bedingungen ermöglicht wird.

(…)

§87c (referring to art. 9(b) of Directive 96/9/EC):

(1) Die Vervielfältigung eines nach Art oder Umfang wesentlichen Teils einer Datenbank ist zulässig

1. (…)

2. zum eigenen wissenschaftlichen Gebrauch, wenn und soweit die Vervielfältigung zu diesem Zweck geboten ist und der wissenschaftliche Gebrauch nicht zu gewerblichen Zwecken erfolgt,

3. für die Benutzung zur Veranschaulichung des Unterrichts, sofern sie nicht zu gewerblichen Zwecken erfolgt.

In den Fällen der Nummern 2 und 3 ist die Quelle deutlich anzugeben.

(…)

### Italy

Copyright and database protection in Italy is provided for by Art. 2575 ss. of the Codice Civile and in the “Legge 22 Aprile 1941 n. 633 Protezione del diritto d’autore e di altri diritti connessi al suo esercizio“ (Interlex 2008). The exceptions and limitations provided for in Directive 2001/29/EC and Directive 96/9/EC have been transformed into the following articles:

Art. 68 (referring to article 5.2.c of the Directive 2001/29/EC):

1. E’ libera la riproduzione di singole opere o brani di opere per uso personale dei lettori, fatta a mano o con mezzi di riproduzione non idonei a spaccio o diffusione dell’opera nel pubblico.

2. E’ libera la fotocopia di opere esistenti nelle biblioteche accessibili al pubblico o in quelle scolastiche, nei musei pubblici o negli archivi pubblici, effettuata dai predetti organismi per i propri servizi, senza alcun vantaggio economico o commerciale diretto o indiretto.

3. Fermo restando il divieto di riproduzione di spartiti e partiture musicali, è consentita, nei limiti del quindici per cento di ciascun volume o fascicolo di periodico, escluse le pagine di pubblicità, la riproduzione per uso personale di opere dell’ingegno effettuata mediante fotocopia, xerocopia o sistema analogo.

4. I responsabili dei punti o centri di riproduzione, i quali utilizzino nel proprio ambito o mettano a disposizione di terzi, anche gratuitamente, apparecchi per fotocopia, xerocopia o analogo sistema di riproduzione, devono corrispondere un compenso agli autori ed agli editori delle opere dell’ingegno pubblicate per le stampe che, mediante tali apparecchi, vengono riprodotte per gli usi previsti nel comma 3. La misura di detto compenso e le modalità per la riscossione e la ripartizione sono determinate secondo i criteri posti all’art. 181- ter della presente legge. Salvo diverso accordo tra la SIAE e le associazione delle categorie interessate, tale compenso non può essere inferiore per ciascuna pagina riprodotta al prezzo medio a pagina rilevato annualmente dall’ISTAT per i libri.

Art. 70 (referring to article 5.3.a of the Directive 2001/29/EC):

1. Il riassunto, la citazione o la riproduzione di brani o di parti di opera e la loro comunicazione al pubblico sono liberi se effettuati per uso di critica o di discussione, nei limiti giustificati da tali fini e purché non costituiscano concorrenza all’utilizzazione economica dell’opera; se effettuati a fini di insegnamento o di ricerca scientifica l’utilizzo deve inoltre avvenire per finalità illustrative e per fini non commerciali.

1-bis. È consentita la libera pubblicazione attraverso la rete internet, a titolo gratuito, di immagini e musiche a bassa risoluzione o degradate, per uso didattico o scientifico e solo nel caso in cui tale utilizzo non sia a scopo di lucro. Con decreto del Ministro per i beni e le attività culturali, sentiti il Ministro della pubblica istruzione e il Ministro dell’università e della ricerca, previo parere delle Commissioni parlamentari competenti, sono definiti i limiti all’uso didattico o scientifico di cui al presente comma.

2. Nelle antologie ad uso scolastico la riproduzione non può superare la misura determinata dal regolamento, il quale fissa la modalità per la determinazione dell’equo compenso.

3. Il riassunto, la citazione o la riproduzione debbono essere sempre accompagnati dalla menzione del titolo dell’opera, dei nomi dell’autore, dell’editore e, se si tratti di traduzione, del traduttore, qualora tali indicazioni figurino sull’opera riprodotta.

Art. 71-ter (referring to article 5.3.n of the Directive 2001/29/EC):

1. E’ libera la comunicazione o la messa a disposizione destinata a singoli individui, a scopo di ricerca o di attività privata di studio, su terminali aventi tale unica funzione situati nei locali delle biblioteche accessibili al pubblico, degli istituti di istruzione, nei musei e negli archivi, limitatamente alle opere o ad altri materiali contenuti nelle loro collezioni e non soggetti a vincoli derivanti da atti di cessione o da licenza.

Art. 64-sexies (referring to article 9(b) of Directive 96/9/EC):

1. Non sono soggetti all’autorizzazione di cui all’articolo 64-quinquies da parte del titolare del diritto:

a) l’accesso o la consultazione della banca di dati quando abbiano esclusivamente finalità didattiche o di ricerca scientifica, non svolta nell’ambito di un’impresa, purché si indichi la fonte e nei limiti di quanto giustificato dallo scopo non commerciale perseguito. Nell’ambito di tali attività di accesso e consultazione, le eventuali operazioni di riproduzione permanente della totalità o di parte sostanziale del contenuto su altro supporto sono comunque soggette all’autorizzazione del titolare del diritto;

b) l’impiego di una banca di dati per fini di sicurezza pubblica o per effetto di una procedura amministrativa o giurisdizionale.

2. Non sono soggette all’autorizzazione dell’autore le attività indicate nell’articolo 64-quinquies poste in essere da parte dell’utente legittimo della banca di dati o di una sua copia, se tali attività sono necessarie per l’accesso al contenuto della stessa banca di dati e per il suo normale impiego; se l’utente legittimo è autorizzato ad utilizzare solo una parte della banca di dati, il presente comma si applica unicamente a tale parte.

3. Le clausole contrattuali pattuite in violazione del comma 2 sono nulle ai sensi dell’articolo 1418 del codice civile.

4. Conformemente alla Convenzione di Berna per la protezione delle opere letterarie e artistiche, ratificata e resa esecutiva con legge 20 giugno 1978, n. 399, le disposizioni di cui ai commi 1 e 2 non possono essere interpretate in modo da consentire che la loro applicazione arrechi indebitamente pregiudizio al titolare del diritto o entri in conflitto con il normale impiego della banca di dati.

Art. 102-ter (referring to article 9(b) of Directive 96/9/EC):

1. L’utente legittimo della banca di dati messa a disposizione del pubblico non può arrecare pregiudizio al titolare del diritto d’autore o di un altro diritto connesso relativo ad opere o prestazioni contenute in tale banca.

2. L’utente legittimo di una banca di dati messa in qualsiasi modo a disposizione del pubblico non può eseguire operazioni che siano in contrasto con la normale gestione della banca di dati o che arrechino un ingiustificato pregiudizio al costitutore della banca di dati.

3. Non sono soggette all’autorizzazione del costitutore della banca di dati messa per qualsiasi motivo a disposizione del pubblico le attività di estrazione o reimpiego di parti non sostanziali, valutate in termini qualitativi e quantitativi, del contenuto della banca di dati per qualsivoglia fine effettuate dall’utente legittimo. Se l’utente legittimo è autorizzato ad effettuare l’estrazione o il reimpiego solo di una parte della banca di dati, il presente comma si applica unicamente a tale parte.

4. Le clausole contrattuali pattuite in violazione dei commi 1, 2 e 3 sono nulle.

### Norway

Copyright and database protection in Norway is provided for by the “Lov av 12. mai 1961 om opphavsrett til åndverk m.v. (åndverksloven)” (Lovdata 2013). The exceptions and limitations provided for in Directive 2001/29/EC und Directive 96/9/EC have been transformed into national law in form of extended collective licenses (“avtalelisenser”). An extended collective license is an agreement between a qualified user (archive, libraries and others) and an officially recognised collecting society which represents “an essential part of authors whose works are used in Norway” (§ 38a). The following articles are particularly relevant:

§ 16. (referring to paragraph 5.2.c and 5.3.n of Directive 2001/29/EC):

Kongen kan gi regler om rett for arkiv, bibliotek, museer og undervisnings- og forskningsinstitusjoner til å fremstille eksemplar av verk for konserverings- og sikringsformål og andre særskilte formål. Bestemmelsen gjelder ikke for ervervsmessig bruk.

Kongen kan gi regler om at arkiv, bibliotek, museer og undervisningsinstitusjoner ved hjelp av terminaler i egne lokaler, kan gjøre verk i samlingene tilgjengelig for enkeltpersoner når det skjer til forskningsformål eller private studieformål.

(The institutions to which the regulations apply are specified in § 1-1 forskrift til åndverksloven as “arkiv under arkivverket, kommunale og fylkeskommunale arkiv, bibliotek under universiteter og høgskoler og andre vitenskapelige og faglige bibliotek som drives av det offentlige, fylkesbibliotekene og folkebibliotekene samt offentlige museer og museer som mottar offentlige tilskudd” [archives of instutional archives, communal and provincial archives, university and highschool libraries and other publicly organised scientific or specialised libraries, provincial and public libraries including public museums and publicly funded museums]. The authorization to make available protected works within the premises of these institutions is specified in § 1-9 forskrift til åndverksloven. Teaching and research institutions can be authorised by the state to produce copies in other formats than the original one [§ 1-4 forskrift til åndverksloven]).

§ 16a. (referring to paragraph 5.2.c and 5.3.n of Directive 2001/29/EC):

Arkiv, bibliotek og museer som angitt i § 16 første ledd kan fremstille eksemplar av utgitte verk i samlingene og gjøre slike verk tilgjengelig for allmennheten når betingelsene for avtalelisens etter § 36 første ledd er oppfylt.

§ 36. (concerning extended collective licenses)

Når det foreligger avtale med organisasjon som nevnt i § 38a som tillater slik bruk av verk som nevnt i §§ 13b, 14, 16a, 17b, 30, 32 og 34, har bruker som omfattes av avtalen, overfor rettighetshaver som ikke er omfattet, rett til på samme område og på samme måte å utnytte verk av samme art som dem avtalen gjelder (avtalelisens). Bestemmelsen gjelder bare for bruk som skjer i samsvar med det avtalen fastsetter. Bestemmelsen gjelder ikke i forhold til kringkastingsforetaks rettigheter i sine sendinger.

§ 38a. (concerning extended collective licenses)

Avtale som skal ha virkning som nevnt i § 36 første ledd, må inngås av organisasjon som på området representerer en vesentlig del av opphavsmennene til verk som brukes i Norge, og som er godkjent av departementet. For bruk på nærmere angitte områder kan Kongen bestemme at den organisasjon som godkjennes, må være en felles organisasjon for de berørte rettighetshavere.

Kongen kan gi nærmere bestemmelser om kontroll med organisasjoner og fond som mottar vederlag til videre fordeling.

### Sweden

Copyright and database protection in Sweden is provided for by the “Lag (1960:729) om upphovsrätt till litterära och künstnärliga verk“ (Sveriges Riksdag 1960). The exceptions and limitations provided for in Directive 2001/29/EC and Directive 96/9/EC have been transformed into the following articles:

16 § (referring to paragraph 5.2.c of Directive 2001/29/EC):

De arkiv och bibliotek som avses i tredje och fjärde styckena har rätt att framställa exemplar av verk, dock inte datorprogram,

1. för bevarende-, kompletterings- eller forskningsändamål,

2. för att tillgodose lånesökandes önskemål om enskilda artiklar eller korta avsnitt eller om material som av säkerketsskäl inte bör lämnas ut ioriginal, eller

3. för användning i läsapparater.

(…)

Rätt till exemplarframställning och spridning enligt denna paragraf har

1. de statliga och kommunala arkivmyndigheterna,

2. Statens ljud- och bildarkiv,

3. de vetenskapeliga bibliotek och fackbibliotek som drivs av det allmänna samt

4. folkbiblioteken.

(…)

In addition, libraries and certain other institutions may make their collection available to the public on the basis of a so called extended collective licence (“avtalelicens”, 42 a–42 f §§). An extended collective licence is an agreement between a qualified user (the library) and a national collecting society which represents a considerable number of Swedish right-holders that oversee certain uses of protected works. The agreement applies not only to the use of works of represented authors but also to the use of any other work of the same kind:

“En avtalelicens som avses i 42 b–42 f §§ gäller för utnyttjande av verk på visst sätt, när ett avtal har ingåtts om utnyttjande av verk påsådant sätt med en organisation som företräder ett flertal svenska upphovsmän på området. Avtalelicensen ger använderen rätt att utnytta verk av det slag som avses med avtalet trots att verkens upphovsmän inte företräds av organisationen” (42a §).

### Switzerland

Copyright and database protection in Switzerland is provided for by the “Bundesgesetz über das Urheberrecht und verwandte Schutzrechte (Urheberrechtsgesetz, URG) vom 9. Oktober 1992“ (Bundesversammlung der Schweizerischen Eidgenossenschaft 1992). As Switzerland is neither part of the EU nor the EEA, EU-Directives do not apply and they do not have to be transformed into national Swiss law. However, Switzerland’s national copyright law provides a series of regulations that seek the same objectives as the exceptions and limitations provided for in Directive 2001/29/EC and Directive 96/9/EC.

Switzerland does not provide for any database protection. Databases are protected only as far as they qualify as works in the sense of copyright. A specific database protection in form of a sui-generis-right does not exist.

Swiss copyright law provides no exceptions and limitations for scientific research. However, it provides exceptions for reproduction of copyrighted works within public libraries, educational institutions, museums and archives as well as exceptions for the private use of copyrighted works. As Swiss law applies, the concept of “private” includes the internal use in private entities and institutions, including commercial organisations and enterprises. Consequently, the exceptions for private use apply to a much wider set of subjects than in other countries. The regulations are to be found in the following articles:

Art. 19 Verwendung zum Eigengebrauch

^1^Veröffentlichte Werke dürfen zum Eigengebrauch verwendet werden. Als Eigengebrauch gilt:

a. jede Werkverwendung im persönlichen Bereich und im Kreis von Personen, die unter sich eng verbunden sind, wie Verwandte und Freunde;

b. jede Werkverwendung der Lehrperson in der Klasse;

c. das Vervielfältigen von Werkexemplaren in Betrieben, öffentlichen Verwaltungen, Institutionen, Kommissionen und ähnlichen Einrichtungen für die interne Information und Kommunikation.

^2^Wer zum Eigengebrauch berechtigt ist, darf unter Vorbehalt von Absatz 3 die dazu erforderlichen Vervielfältigungen auch durch Dritte herstellen lassen; als Dritte im Sinne dieses Absatzes gelten auch Bibliotheken, andere öffentliche Institutionen und Geschäftsbetriebe, die ihren Benützern und Benützerinnen Kopiergeräte zur Verfügung stellen.

^3^Ausserhalb des privaten Kreises nach Absatz 1 Buchstabe a sind nicht zulässig:

a. die vollständige oder weitgehend vollständige Vervielfältigung im Handel erhältlicher Werkexemplare;

b. die Vervielfältigung von Werken der bildenden Kunst;

c. die Vervielfältigung von graphischen Aufzeichnungen von Werken der Musik;

d. die Aufnahme von Vorträgen, Aufführungen oder Vorführungen eines Werkes auf Ton-, Tonbild- oder Datenträger.

(…)

Art. 24 Archivierungs- und Sicherungsexemplare

(…)

^1bis^ Öffentlich zugängliche Bibliotheken, Bildungseinrichtungen, Museen und Archive dürfen die zur Sicherung und Erhaltung ihrer Bestände notwendigen Werkexemplare herstellen, sofern mit diesen Kopien kein wirtschaftlicher oder kommerzieller Zweck verfolgt wird.

(…)

### United Kingdom

Copyright and database protection in the UK is provided for by the “Copyright, Designs and Patents Act 1988“ (CDPA) and in the “The Copyright and Rights in Databases Regulations 1997” (United Kingdom Parliament 1988). The exceptions and limitations provided for in Directive 2001/29/EC and Directive 96/9/EC have been transformed into the following articles:

Section 29 CDPA (referring to article 5.3.a and 5.3.n of Directive 2001/29/EC):

(1) Fair dealing with a work for the purposes of research for a non-commercial purpose does not infringe any copyright in the work provided that it is accompanied by a sufficient acknowledgement.

(1B) No acknowledgement is required in connection with fair dealing for the purposes mentioned in subsection (1) where this would be impossible for reasons of practicality or otherwise.

(1C) Fair dealing with a work for the purposes of private study does not infringe any copyright in the work.

(3) Copying by a person other than the researcher or student himself is not fair dealing if—

(a) in the case of a librarian, or a person acting on behalf of a librarian, that person does anything which regulations under section 42A (copying by librarians: single copies of published works), or

(b) in any other case, the person doing the copying knows or has reason to believe that it will result in copies of substantially the same material being provided to more than one person at substantially the same time and for substantially the same purpose.

Section 29A CDPA (referring to article 5.2.c of Directive 2001/29/EC):

(1) The making of a copy of a work by a person who has lawful access to the work does not infringe copyright in the work provided that—

(a) the copy is made in order that a person who has lawful access to the work may carry out a computational analysis of anything recorded in the work for the sole purpose of research for a non-commercial purpose, and

(b) the copy is accompanied by a sufficient acknowledgement (unless this would be impossible for reasons of practicality or otherwise).

(2) Where a copy of a work has been made under this section, copyright in the work infringed if—

(a) the copy is transferred to any other person, except where the transfer is authorised by the copyright owner, or

(b) the copy is used for any purpose other than that mentioned in subasection (1)(a), except where the use is authorised by the copyright owner.

(3) If a copy made under this section is subsequently dealt with—

(a) it is to be treated as an infringing copy for the purposes of that dealing, and

(b) if that dealing infringes copyright, it is to be treated as an infringing copy for all subsequent purposes.

(4) In subsection (3) “dealt with” means sold or let for hire, or offered or exposed for ale or hire.

(5) To the extent that a term of a contract purports to prevent or restrict the making of a copy which, by virtue of this section, would not infringe copyright, that term is unenforceable”.

Section 38 CDPA (referring to article 5.2.c of Directive 2001/29/EC):

(1) The librarian of a prescribed library may, if the prescribed conditions are complied with, make and supply a copy of an article in a periodical without infringing any copyright in the text, in any illustrations accompanying the text or in the typographical arrangement.

(2) The prescribed conditions shall include the following—

(a) that copies are supplied only to persons satisfying the librarian that they require them for the purposes of—

(i) research for a non-commercial purpose, or

(ii) private study, and will not use them for any other purpose;

(b) that no person is furnished with more than one copy of the same article or with copies of more than one article contained in the same issue of a periodical; and

(c) that persons to whom copies are supplied are required to pay for them a sum not less than the cost (including a contribution to the general expenses of the library) attributable to their production.

Section 39 CDPA (referring to article 5.2.c of Directive 2001/29/EC):

(1) The librarian of a prescribed library may, if the prescribed conditions are complied with, make and supply from a published edition a copy of part of a literary, dramatic or musical work (other than an article in a periodical) without infringing any copyright in the work, in any illustrations accompanying the work or in the typographical arrangement.

(2) The prescribed conditions shall include the following—

(a) that copies are supplied only to persons satisfying the librarian that they require them for the purposes of—

(i) research for a non-commercial purpose, or

(ii) private study,

and will not use them for any other purpose;

(b) that no person is furnished with more than one copy of the same material or with a copy of more than a reasonable proportion of any work; and

(c) that persons to whom copies are supplied are required to pay for them a sum not less than the cost (including a contribution to the general expenses of the library) attributable to their production.

Section 40 CDPA (referring to article 5.2.c, 5.3.a and 5.3.n of Directive 2001/29/EC):

(1) Regulations for the purposes of sections 38 and 39 (copying by librarian of article or part of published work) shall contain provision to the effect that a copy shall be supplied only to a person satisfying the librarian that his requirement is not related to any similar requirement of another person.

(2) The regulations may provide—

(a) that requirements shall be regarded as similar if the requirements are for copies of substantially the same material at substantially the same time and for substantially the same purpose; and

(b) that requirements of persons shall be regarded as related if those persons receive instruction to which the material is relevant at the same time and place.

Section 42A CDPA (referring to article 5.3.a und 5.3.n of Dirctive 2001/29 EC):

(1) A librarian of a library which is not conducted for profit may, if the conditions in subsection (2) are met, make and supply a single copy of—

(a) one article in any one issue of a periodical, or

(b) a reasonable proportion of any other published work, without infringing copyright in the work.

(2) The conditions are—

(a) a copy is supplied in response to a request from a person who has provided the librarian with a declaration in writing which includes the information set out in subsection (3), and

(b) the librarian is not aware that the declaration is false in a material particular.

(3) The information which must be included in the declaration is—

(a) the name of the person who requires the copy and the material which that person requires,

(b) s statement that the person has not previously been supplied with a copy of that material by any library,

(c) a statement that the person requires the copy for the purpose of research for a non-commercial purpose or private study, will use it only for those purposes and will not supply the copy to any other person, and

(d) a statement that to the best of the person’s knowledge, no other person with whom the person works or studies has made, or intends to make, at or about the same time as the person’s request, a request for substantially the same material for substantially the same purpose.

Section 50 D CDPA (referring to Article 9(b) of Directive 96/9/EC):

(1) It is not an infringement of copyright in a database for a person who has a right to use the database or any part of the database, (whether under a licence to do any of the acts restricted by the copyright in the database or otherwise) to do, in the exercise of that right, anything which is necessary for the purposes of access to and use of the contents of the database or of that part of the database.

(2) Where an act which would otherwise infringe copyright in a database is permitted under this section, it is irrelevant whether or not there exists any term or condition in any agreement which purports to prohibit or restrict the act (such terms being, by virtue of section 296B, void).

### Summary of national regulations

**Table 1.** Summary of national regulations.

	D 2001/29 Art. 5.2.c	D 2001/29 Art. 5.3.a	D 2001/29 Art. 5.3.n	D 96/9 Art. 9(b)
**Denmark**	allowed through extended collective license	allowed through extended collective license	legal license	no provision
**France**	legal license	legal license (except for school books)	no provision	no provision
**Germany**	legal license (restricted)	legal license (except for school books)	allowed within the premises, if no other data use agreement	legal license (except for commercial use)
**Italy**	legal license	legal license (restricted)	allowed within the premises, if no other data use agreement	legal license (except for commercial use)
**Norway**	legal license	no provision	allowed through extended collective license	no database protection
**Sweden**	legal license	no provision	allowed through extended collective license	no provision
**Switzerland**	legal license	no provision	no provision	no database protection
**United Kingdom**	legal license (restricted)	legal license (linked to “fair dealing”)	legal license (linked to “fair dealing”)	legal license

A legal license refers to the use of protected works allowed by law, normally linked to a levy. Legal licenses supersede individual data use agreements. Extended collective licenses are agreements between a qualified user (e.g. a library) and a national collecting society which represents a considerable number of national right-holders. The figures in the first line refer to the following provisions: Directive 2001/29/EC of the European Parliament and of the Council of 22 May 2001 on the harmonisation of certain aspects of copyright and related rights in the information society. Article 5.2: Member States may provide for exceptions or limitations to the reproduction right provided for in Article 2 in the following cases: (c) in respect of specific acts of reproduction made by publicly accessible libraries, educational establishments or museums, or by archives, which are not for direct or indirect economic or commercial advantage Article 5.3. Member States may provide for exceptions or limitations to the rights provided for in Articles 2 and 3 in the following cases: (a) use for the sole purpose of illustration for teaching or scientific research, as long as the source, including the author’s name, is indicated, unless this turns out to be impossible and to the extent justified by the non-commercial purpose to be achieved. (n) use by communication or making available, for the purpose of research or private study, to individual members of the public by dedicated terminals on the premises of establishments referred to in paragraph 2(c) of works and other subject-matter not subject to purchase or licensing terms which are contained in their collections Directive 2001/29/EC of the European Parliament and of the Council of 11 March 1996 on the legal protection of databases. Article 9 Exceptions to the sui generis right: Member States may stipulate that lawful users of a database which is made available to the public in whatever manner may, without the authorization of its maker, extract or re-utilize a substantial part of its contents: (b) in the case of extraction for the purposes of illustration for teaching or scientific research, as long as the source is indicated and to the extent justified by the non-commercial purpose to be achieved

## Conclusion

This review illustrates that national provisions in Europe on copyright and database protection regarding exceptions and limitations for research purposes differ not only in certain details but in substance. There is no consistency among national legislations despite Directive 2001/29/EC that aims to achieve harmonisation. In none of the regulations presented here is there a general exception or limitation to the copyright protection for the use of works for research purposes. In some countries, there are exceptions for some particular uses in the sector of scientific research. In Scandinavian countries, there is an exception linked to extended collective licence schemes. In the United Kingdom there is a very detailed exception for research purposes linked to a fair-dealing-clause. Exceptions to the sui-generis-database-protection are even more varied (Table 1).

Most national regulations seem to be oriented to a rather outdated concept of scientific work. Provisions that link copyright exceptions to the premises of certain institutions are incompatible with the current nature of scientific collaboration among institutions. Provisions that allow for the printing of copies but not for the storage on digital devices (as is the case in Germany), or that forbid the sharing of documents (as is the case in the recently introduced section 42A of the British CPDA), impair the efficiency of scientific work and confine researchers to outdated working methods.

Regulations that link exceptions and limitations to idiosyncratic definitions of works (for example, in Germany, Art. 53a par. 1 UrhG establishes that the exception for library copies is allowed if the same work cannot be purchased in a library or via the internet but is not allowed if the item can be purchased) make modern research techniques such as automated text generation and data mining difficult. It is not possible to search or analyse automatically a body of documents if there is no general rule about what can and cannot be done with all the documents. In the interests of efficient scientific research, exceptions and limitations of copyright and database protection should, therefore, apply to every object that is of interest to researchers.

There is no longer a clear distinction between commercial and non-commercial research (Hagedorn et al. 2011). Public-private partnerships and sponsored research render this distinction obsolete. A research institution that makes earnings from selling products or services cannot pretend to do non-commercial research. No institution can guarantee that the results of its research will not partly be commercialised by third persons. Regulations that restrict exceptions and limitations to the re-use of works for non-commercial research purposes misjudge the reality of science. The distinction is neither applicable nor useful and should be abandoned.

The emergence of an integrated system for the management of biodiversity knowledge will be hampered by current copyright and database protection. This hurdle should be removed by unifying exceptions and limitations for research purposes in a binding, Europe-wide regulation.
